# Gut microbiota dysbiosis aggravates sepsis-induced lung injury by promoting neutrophil extracellular traps and suppressing host integrin defense

**DOI:** 10.3389/fmicb.2025.1699748

**Published:** 2026-01-09

**Authors:** Zhiyong Zhao, Bingjie Wu

**Affiliations:** 1 Department of Emergency Medicine, Shanxi Bethune Hospital, Shanxi Academy of Medical Sciences, Third Hospital of Shanxi Medical University, Tongji Shanxi Hospital, Taiyuan, China; 2 Department of Infection Diseases, First Hospital of Shanxi Medical University, Taiyuan, China

**Keywords:** acute lung injury, gut microbiota, integrin alpha M, integrin beta 2, neutrophil extracellular traps, sepsis

## Abstract

**Background:**

The gut-lung axis is central to systemic inflammatory regulation, but the mechanisms by which gut microbiota dysbiosis aggravates sepsis-induced acute lung injury (ALI), particularly through neutrophil extracellular traps (NETs) and integrin signaling, remain unclear. Given the critical need for microbiota-based therapeutic strategies, this study investigates the mechanistic link between gut microbiota, NET formation, and pulmonary endothelial barrier dysfunction.

**Methods:**

Using a cecal ligation and puncture (CLP) sepsis model, control, sepsis, and fecal microbiota transplantation (FMT) groups were compared. Lung injury was assessed via histopathology, wet/dry weight ratios, and bronchoalveolar lavage fluid (BALF) analysis. High-throughput RNA sequencing (GO/KEGG/PPI) identified key targets, validated by lentiviral knockdown/overexpression of ITGAM and ITGB2 *in vivo* and *in vitro* [mouse pulmonary microvascular endothelial cells (MPMECs) and neutrophil co-cultures]. NETs were quantified by MPO-DNA ELISA and immunofluorescence.

**Results:**

CLP-induced sepsis triggered severe pulmonary edema, neutrophil infiltration, and NET accumulation, alongside downregulation of ITGAM/ITGB2 and tight junction proteins (β-catenin/ZO-1/VE-cadherin). FMT reduced NETs by 58% (*p* < 0.001) and restored endothelial barrier integrity. Transcriptomics revealed ITGAM/ITGB2 as central nodes in neutrophil activation and integrin pathways. *In vitro*, NET exposure increased endothelial permeability (3.1-fold FITC-dextran flux, *p* < 0.01) and IL-6/TNF-α secretion, while ITGAM/ITGB2 overexpression reversed these effects. Conversely, integrin silencing abolished FMT’s protection, exacerbating ALI.

**Conclusion:**

We unveil a novel gut microbiota-NET-integrin axis in sepsis-induced ALI, where microbial dysbiosis promotes NET-mediated suppression of ITGAM/ITGB2, leading to endothelial barrier failure. Our findings position FMT and integrin modulation as promising strategies to mitigate pulmonary vascular dysfunction, advancing the therapeutic potential of microbiota-targeted interventions in critical care.

## Graphical abstract

**Figure:**
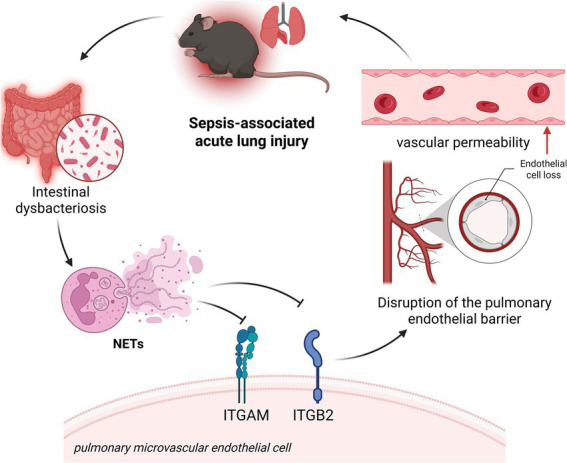
Schematic representation of the molecular mechanism by which gut microbiota dysbiosis triggers the formation of NETs, leading to the downregulation of ITGAM and ITGB2, disruption of pulmonary endothelial barrier integrity, and exacerbation of sepsis-induced ALI.

## Introduction

Sepsis, a systemic inflammatory syndrome triggered by infection, continues to exhibit high incidence and mortality rates, posing a serious threat to patient survival (Dartiguelongue, 2020; Wang W. et al., 2024; Wang Z. et al., 2024). Among the various organ systems affected, acute lung injury (ALI) is one of the most frequently involved and represents a leading cause of sepsis-related death (Zhang et al., 2024; Miyashita et al., 2016). Clinically, ALI is characterized by increased permeability of the alveolar epithelium and pulmonary capillary endothelium, extensive infiltration of inflammatory cells, and impaired alveolar gas exchange (Salomão et al., 2019, Prado et al., 2024). Current treatment strategies for ALI are primarily supportive in nature, with a lack of effective targeted therapies (Li F. et al., 2024; Li X. et al., 2024; Zhang F. et al., 2023; Zhang H. et al., 2023). Growing evidence indicates that endothelial barrier dysfunction is central to the pathogenesis of ALI, underscoring the importance of clarifying the molecular mechanisms driving pulmonary endothelial barrier disruption (Fang et al., 2025; Zhong et al., 2024). In recent years, studies have increasingly emphasized the roles of immune cells in pulmonary inflammation, particularly the dual effects of neutrophil activation and their inflammatory mediators in aggravating lung injury (Zou et al., 2023; Wang F. et al., 2022; Wang Z. et al., 2022).

Neutrophil extracellular traps (NETs) are web-like structures released by activated neutrophils, consisting mainly of deoxyribonucleic acid (DNA) and proteolytic enzymes, and function as a frontline antimicrobial defense (Chen et al., 2021; Kiwit et al., 2024). However, excessive NET formation has been linked to the pathogenesis of several inflammatory diseases, including sepsis and ALI (Zou et al., 2024; Zhang et al., 2022). In the context of sepsis, NETs tend to accumulate within the pulmonary microvasculature, leading to endothelial injury, increased vascular permeability, and exacerbated tissue edema, thereby amplifying local inflammatory responses (Zhu et al., 2023; Fei et al., 2024). NETs disrupt endothelial tight junctions and promote pro-inflammatory cytokine release, ultimately compromising pulmonary vascular barrier integrity (Zhang F. et al., 2023; Zhang H. et al., 2023; Meegan et al., 2018). Moreover, NETs interact with other immune components such as platelets and the complement system, contributing to the modulation of the pulmonary immune microenvironment (Huang et al., 2022; Liu et al., 2023). Targeting aberrant NET activation and its downstream effects on lung tissue thus represents a promising therapeutic avenue for mitigating sepsis-induced ALI (McDonald et al., 2017).

Integrins are a crucial class of transmembrane glycoproteins that mediate cell–cell adhesion, signal transduction, and immune regulation (Schymeinsky et al., 2007; Khan et al., 2018). Integrin alpha M (ITGAM) and integrin beta 2 (ITGB2) together constitute the CD11b/CD18 heterodimer, which plays a pivotal role in neutrophil migration, activation, and phagocytic function (Fagerholm et al., 2006; Gottschalk et al., 2022). Previous studies have demonstrated that ITGAM and ITGB2 act as molecular bridges facilitating neutrophil-endothelial adhesion and immune activation, and are also involved in regulating the transendothelial migration of inflammatory cells (Zhou et al., 2013; Chidlow et al., 2010). However, the expression dynamics and biological significance of these integrins in sepsis-induced ALI remain insufficiently characterized (Wang W. et al., 2024; Wang Z. et al., 2024; Yuan et al., 2012). Downregulation of ITGAM and ITGB2 may compromise endothelial responsiveness to injurious stimuli, exacerbating barrier dysfunction and amplifying inflammation (Hu et al., 2023; Meegan et al., 2018). Notably, integrins may serve as critical regulatory nodes through which NETs exert their effects on the endothelium, thereby modulating both barrier integrity and inflammatory signaling. Clarifying the specific roles of ITGAM and ITGB2 within this context is therefore of considerable importance (Xie B. et al., 2024; Xie R. et al., 2024; Hong et al., 2021).

Recent advances in the study of the “gut-lung axis” have underscored the far-reaching immunomodulatory effects of the gut microbiota on pulmonary homeostasis (Zhang et al., 2020; Druszczynska et al., 2024). Under physiological conditions, the gut microbiome contributes to immune balance in the lungs through microbial metabolites, immune cell education, and maintenance of mucosal barriers (Verma et al., 2024; Ni et al., 2023). In contrast, dysbiosis can induce systemic inflammation and indirectly intensify pulmonary immune responses (Hung and Matute-Bello, 2022; Tang et al., 2024). Accumulating evidence suggests that alterations in gut microbiota composition significantly influence neutrophil activation and the formation of NETs (Li G. et al., 2023; Li Z. et al., 2023; Vong et al., 2014). For instance, microbial-associated molecular patterns (MAMPs) derived from a dysbiotic gut may activate Toll-like receptor (TLR) pathways, leading to excessive NET release and aggravation of distal lung injury (Alsabani et al., 2022; Hawez et al., 2021). Moreover, microbial metabolites such as short-chain fatty acids (SCFAs) modulate immune cell function and attenuate inflammatory responses (Mann et al., 2024; Wang et al., 2017). These observations suggest that gut microbiota (GM) may influence ALI severity by regulating NET formation and integrin expression, thereby affecting the integrity of the pulmonary endothelial barrier (Xuan et al., 2024; Wang F. et al., 2022; Wang Z. et al., 2022). Nonetheless, the mechanistic interplay between the gut-lung axis, NETs, and integrin-mediated signaling remains poorly elucidated (Xie B. et al., 2024; Xie R. et al., 2024; Song et al., 2021).

Against this backdrop, the present study investigates whether GM dysbiosis exacerbates sepsis-induced ALI by promoting NET formation and suppressing the expression of the integrins ITGAM and ITGB2, thereby disrupting pulmonary endothelial barrier integrity. A cecal ligation and puncture (CLP) model was used to induce sepsis, and fecal microbiota transplantation (FMT) served to restore microbial homeostasis. High-throughput RNA sequencing, Gene Ontology (GO) and Kyoto Encyclopedia of Genes and Genomes (KEGG) enrichment analyses, as well as protein–protein interaction (PPI) network construction, were integrated to identify key molecular targets. Both *in vivo* and *in vitro* assays were conducted to systematically explore the interaction between NETs and ITGAM/ITGB2 in modulating endothelial barrier function. This study aims to delineate the mechanistic axis linking the GM, NETs, and integrin signaling in the context of sepsis-induced ALI, thereby enhancing our understanding of inter-organ communication and immune regulation. Importantly, ITGAM and ITGB2 may emerge as promising therapeutic targets for mitigating sepsis-induced ALI, while microbiota-based interventions such as FMT offer translational potential as adjunctive therapeutic strategies.

## Materials and methods

### High-throughput sequencing and data analysis

Lung tissues from three mice per group (Control, Model, and FMT; *n* = 9 total) were collected for high-throughput RNA sequencing. Total RNA was extracted using the Total RNA Isolation Kit (12183555, Invitrogen, United States) and quantified by ultraviolet spectrophotometry (BioSpectrometer basic, Eppendorf, United States). RNA integrity was confirmed by agarose gel electrophoresis. High-quality RNA was reverse-transcribed for library preparation and sequenced on the Illumina NextSeq 500 platform. Raw image files were converted to reads by base calling, and sequencing adapters and low-quality reads were removed using cutadapt to obtain clean reads. Clean reads were aligned to the mouse reference genome using Hisat2, and gene expression levels were quantified in R.

Differentially expressed genes (DEGs) were identified using the “limma” package (|log_2_FC| >2, adjusted *p* < 0.01). Venn diagrams and volcano plots were generated via the Xiantao academic platform,^1^ and expression heatmaps were produced using the “pheatmap” package in R.

### GO and KEGG pathway enrichment analysis

GO and KEGG enrichment analyses of the identified DEGs were performed using the Xiantao academic platform (see text footnote 1). GO analysis encompassed biological processes (BP), cellular components (CC), and molecular functions (MF). Pathways with *p* < 0.05 were considered significant. KEGG pathway analysis was conducted using the same threshold, and results were visualized with the “ggplot2” package. Enriched pathways related to adipose browning and metabolic regulation were identified based on functional relevance and supporting literature.

### PPI network analysis

Potential therapeutic targets identified from the DEGs were imported into the STRING database,^2^ with *Mus musculus* specified as the organism. The interaction confidence score was set to 0.700, and unconnected nodes were removed. The resulting network elucidated the interaction landscape of candidate targets in sepsis-induced ALI. Interaction data were downloaded, and the degree of connectivity for each protein was quantified and visualized using R.

### Isolation and culture of mouse pulmonary microvascular endothelial cells and neutrophils

Mouse pulmonary microvascular endothelial cells (MPMECs) were isolated using magnetic-activated cell sorting (MACS). Lung tissues from 8-week-old male C57BL/6J mice were dissociated into single-cell suspensions with a mouse lung dissociation kit (130-095-927, Miltenyi Biotec, Japan). A negative selection strategy was applied using CD45 (130-052-301), CD90.2 (130-121-278), and CD326 (130-105-958) microbeads to remove non-endothelial cells. The resulting CD45^−^ CD90.2^−^ CD326^−^ fraction was collected as MPMECs. Cells were seeded into gelatin-coated T25 flasks and maintained in DMEM/F12 supplemented with 10% fetal bovine serum (10099141, Gibco, United States), 1% endothelial cell growth supplement (SC1052, ScienCell, United States), and 1% penicillin–streptomycin (BC-CE-007, Biochannel, China) at 37 °C with 5% CO_2_. Medium was replaced every 2–3 days, and cells were passaged at 80–90% confluence. Third-passage cells were used for subsequent experiments.

Neutrophils were isolated from the peritoneal cavity following euthanasia. Five milliliters of pre-chilled phosphate-buffered saline (PBS, 10010023, Gibco, United States) were injected intraperitoneally, and the exudate was collected. The suspension was centrifuged at 400 g for 10 min (ambient temperature), and the cell pellet was resuspended in PBS and layered over Lympholyte-M (CL5035, Cedarlane, Canada) for density-gradient centrifugation at 500 g for 30 min (ambient temperature, no brake). The neutrophil-rich layer was collected, and residual red blood cells were lysed using BD Pharm Lyse buffer (555899, BD Biosciences, United States). The purified neutrophils were resuspended in pre-chilled RPMI-1640 medium (11875093, Gibco, United States) containing 3% FBS (A5670701, Gibco, United States). Flow cytometry confirmed a purity ≥90% for CD11b^+^Ly6G^+^ cells, with cell viability ≥95% as assessed by trypan blue exclusion.

### Preparation and handling of NETs

Purified neutrophils were resuspended in complete RPMI-1640 medium (11875119, Gibco, United States) and seeded into 6-well plates (354671, Corning, United States) at 1.8 × 10^6^ cells per well. NET formation was induced by stimulation with 50 nM phorbol 12-myristate 13-acetate (PMA; P1585, Sigma-Aldrich, United States) for 4 h at 37 °C under 5% CO_2_. After stimulation, the supernatant was removed and 2 mL of pre-chilled PBS (4 °C) was added. Adherent NETs were gently detached and collected. The suspension was centrifuged at 1,000 × g for 10 min at 4 °C to eliminate cellular debris, followed by a second centrifugation at 20,000 × g for 10 min at 4 °C to pellet NETs. The pellet was resuspended in PBS, and DNA content was measured using the Quant-iT PicoGreen dsDNA Assay Kit (P11496, Thermo Fisher, United States). Fresh NET preparations were used immediately or stored in liquid nitrogen for later experiments.

### Construction and transduction of lentiviral vectors for overexpression and knockdown

Based on the cDNA sequences of murine ITGAM and ITGB2 obtained from the NCBI GenBank database,^3^ three distinct short hairpin RNA (shRNA) sequences targeting different regions were designed, along with a non-targeting control sequence (sh-NC). All sequence information is listed in Supplementary Table S1. The shRNA oligonucleotides were synthesized by GenePharma (Shanghai, China) and cloned into the pLKO.1 lentiviral vector for gene silencing.

The 293 T cell line (CRL-3216, ATCC) was cultured in DMEM (11965092, Gibco, United States) supplemented with 10% FBS, 10 μg/mL streptomycin, and 100 U/mL penicillin, in a humidified incubator maintained at 37 °C with 5% CO_2_ (Heracell™ Vios 160i CR, 51033770, Thermo Scientific^™^). Lentiviral particles were produced using a standard three-plasmid system: packaging plasmid psPAX2, envelope plasmid pMD2.G, and the recombinant pLKO.1-shRNA plasmid was co-transfected into 293 T cells at 80–90% confluence using Lipofectamine 2000 (Invitrogen, 11668500). After 48 h, culture supernatants were harvested, filtered through 0.45 μm membranes, and centrifuged at 50,000 × g for 2 h to concentrate viral particles. Viral titers were determined via flow cytometry based on GFP-positive cell percentages, and titers ≥1 × 10^8^ TU/mL were deemed acceptable.

For overexpression experiments, the pLenti-GFP vector system (Genechem, Shanghai, China) was used, following the same packaging protocol. To generate stable cell lines, cells in logarithmic growth were seeded into 6-well plates at a density of 5 × 10^4^ cells/mL and infected with lentivirus at a multiplicity of infection (MOI) of 10 in the presence of 8 μg/mL polybrene (428175, Sigma-Aldrich, United States). After 48 h of infection, the culture medium was replaced with fresh medium containing 2 μg/mL puromycin (UC0E03, Sigma-Aldrich, United States) for selection over 2 weeks to establish stable transductants (Li et al., 2019).

### Co-culture and experimental grouping

MPMECs were seeded into the lower chamber of 0.4 μm pore-size Transwell inserts (7424, Corning, United States) at 2 × 10^6^ cells/mL and cultured for 48 h to form a confluent endothelial monolayer. Neutrophils were then added to the upper chamber, and the following experimental groups were established:

CON group: Untreated MPMECs co-cultured with neutrophils.
NET group: MPMECs co-cultured with neutrophils stimulated with PMA to induce NET formation (DNA concentration standardized to 10 μg/mL).
NET + oe-NC group: MPMECs transduced with control lentivirus (oe-NC) for 48 h, then co-cultured with PMA-stimulated neutrophils (NET-forming neutrophils).
NET + oe-ITGAM group: MPMECs transduced with ITGAM-overexpressing lentivirus for 48 h, followed by co-culture with PMA-stimulated neutrophils.
NET + oe-ITGB2 group: MPMECs transduced with ITGB2-overexpressing lentivirus for 48 h, followed by co-culture with PMA-stimulated neutrophils.

Lentiviral transduction was performed at an MOI of 20, with 8 μg/mL Polybrene (TR-1003, Sigma-Aldrich, United States) to enhance infection efficiency. After 48 h, the medium was replaced with complete DMEM supplemented with 10% FBS (A5670701, Gibco, United States), and cells were cultured for an additional 24 h prior to NET stimulation (24 h). Following treatment, both cells and culture supernatants were harvested for subsequent analyses.

### Transendothelial electrical resistance measurement

MPMECs were seeded into Transwell inserts (7424, Corning, United States) at 2 × 10^5^ cells/mL and cultured for 48–72 h to form a continuous monolayer. After serum deprivation for 12 h in serum-free DMEM (11965092, Gibco, United States), cells were subjected to designated experimental treatments. Transendothelial electrical resistance (TEER) was continuously monitored using an EVOM2 voltohmmeter (World Precision Instruments, United States). Results were expressed in Ω·cm^2^ and normalized by subtracting the resistance of blank inserts to yield baseline-corrected values.

### Fluorescein isothiocyanate-dextran permeability assay

Following treatment, 1 mg/mL fluorescein isothiocyanate (FITC)-dextran solution (dissolved in serum-free medium; 46944, Sigma-Aldrich, United States) was added to the upper chamber of the Transwell insert, while an equal volume of fresh culture medium was added to the lower chamber. The plates were incubated in the dark at 37 °C in a 5% CO_2_ humidified incubator for 30 min. Subsequently, 100 μL of medium from the lower chamber was transferred to a black 96-well plate (Corning, United States), and fluorescence intensity was measured using a SpectraMax i3 microplate reader (Molecular Devices, United States) at 493 nm excitation and 517 nm emission. FITC-dextran concentrations were calculated from a standard curve, and permeability was expressed as fold change relative to the control group using the formula: (Fluorescence_sample − Fluorescence_blank)/(Fluorescence_control − Fluorescence_blank). Each condition was tested in triplicate, and all experiments were independently replicated three times.

### Quantification of inflammatory cytokines by enzyme-linked immunosorbent assay

Inflammatory cytokines in MPMEC culture supernatants were quantified by enzyme-linked immunosorbent assay (ELISA). After treatment, supernatants were collected and centrifuged at 12,000 × g for 10 min at 4 °C to remove debris. Clarified samples were aliquoted and stored at −80 °C until analysis. Commercial ELISA kits were used to measure interleukin-6 (IL-6; EMC004, Neobioscience, China) and tumor necrosis factor-α (TNF-α; EMC102, Neobioscience, China). The procedure included serial dilution of standards, addition of samples and standards to pre-coated plates, incubation with biotin-conjugated detection antibodies, binding of Horseradish peroxidase (HRP)-labeled streptavidin, and color development using TMB substrate. The reaction was terminated, and absorbance was measured at 450 nm with background correction at 570 nm using a microplate reader (SpectraMax i3, Molecular Devices, United States). Cytokine concentrations were determined from standard curves and expressed in pg/mL. Each sample was analyzed in duplicate, and experiments were independently repeated three times.

### Immunofluorescence staining

For cellular immunofluorescence staining, MPMECs were seeded onto confocal microscope-compatible culture dishes (Nest, China). Following experimental treatments, cells were washed three times with pre-chilled PBS. Fixation was performed using 4% paraformaldehyde (P6148, Sigma-Aldrich, United States) at ambient temperature for 15 min, followed by permeabilization with 0.1% Triton X-100 (648462, Sigma-Aldrich, United States) for 10 min. Cells were then blocked with 5% bovine serum albumin (BSA, 9048-46-8, Sigma-Aldrich, United States) for 1 h at ambient temperature.

The following primary antibodies were applied and incubated overnight at 4 °C: anti-β-catenin (rabbit monoclonal, PA5-19469, Invitrogen, United States), anti-ZO-1 (mouse monoclonal, 33-9100, Invitrogen, United States), anti-ITGAM (rabbit monoclonal, PA5-90724, Invitrogen, United States), and anti-ITGB2 (mouse monoclonal, MA5-15517, Invitrogen, United States). On the following day, cells were washed with PBS and incubated with species-specific secondary antibodies conjugated to Alexa Fluor dyes: Alexa Fluor 488-conjugated anti-rabbit IgG (1:250, A-11008, Invitrogen, United States) and Alexa Fluor 594-conjugated anti-mouse IgG (1:500, A-11005, Invitrogen, United States), for 1 h at ambient temperature in the dark. Nuclei were counterstained with 4′,6-diamidino-2-phenylindole (DAPI) (D9542, Sigma-Aldrich, United States) for 5 min. Slides were mounted and visualized using a Zeiss LSM 880 confocal microscope. Negative controls (without primary antibodies) were included to verify staining specificity.

### Tissue immunofluorescence staining

Paraffin-embedded mouse lung sections were deparaffinized and rehydrated through a graded ethanol series. Endogenous peroxidase activity was quenched with 3% hydrogen peroxide for 30 min at ambient temperature, followed by three PBS washes. Antigen retrieval was performed in 10 mmol/L citrate buffer (pH 6.0) at 94 °C for 10 min. Sections were then blocked with 5% BSA for 1 h at ambient temperature.

Primary antibodies, including rabbit anti-Myeloperoxidase (MPO) (ab208670, Abcam, United Kingdom; 1:100) and mouse anti-CitH3 (#97272, Cell Signaling Technology, United States; 1:800), were incubated overnight at 4 °C. After washing, sections were incubated for 50 min at ambient temperature in the dark with Alexa Fluor 488-conjugated anti-rabbit IgG (A-11008, Invitrogen, United States; 1:500) and Alexa Fluor 594-conjugated anti-mouse IgG (A-11005, Invitrogen, United States; 1:500). Nuclei were counterstained with DAPI. Fluorescence images were captured using a Leica TCS SP8 confocal microscope (Germany), and quantification was performed with ImageJ software.

### Experimental animals and grouping

Specific pathogen-free (SPF) male C57BL/6J mice (6–8 weeks old, 20–24 g) were obtained from Beijing Vital River Laboratory Animal Technology Co., Ltd. Animals were maintained under controlled conditions (22 ± 1 °C, 50% ± 5% humidity) with a 12-h light/dark cycle (08:00–20:00) and free access to food and water. The experimental protocol was reviewed and approved by the Institutional Animal Care and Use Committee, and all procedures were conducted in accordance with the Guide for the Care and Use of Laboratory Animals.

After a 7-day acclimatization period, the mice were randomly assigned to eight groups (*n* = 6 per group) as follows: Control group: healthy control; Model group: disease model; FMT group: fecal microbiota transplantation; FMT + Saline group: fecal microbiota transplantation plus saline control; FMT + DNase group: fecal microbiota transplantation with DNase treatment; FMT + sh-NC group: fecal microbiota transplantation with non-targeting shRNA control; FMT + sh-ITGAM group: fecal microbiota transplantation with ITGAM knockdown; FMT + sh-ITGB2 group: fecal microbiota transplantation with ITGB2 knockdown. The euthanasia methods for these groups at the experimental endpoint are described in the following section.

### Euthanasia

At the end of the experimental period, mice were euthanized by one of the following two methods, both compliant with the AVMA Guidelines for the Euthanasia of Animals:

Carbon dioxide asphyxiation: Mice were placed in a sealed chamber, and CO_2_ was introduced using a gradual-fill method at a displacement rate of 20–30% of the chamber volume per minute. Death was confirmed by ensuring the absence of a foot-pinch reflex followed by cervical dislocation.
Anesthetic overdose for terminal procedure: On day 10 post-operation, mice were deeply anesthetized with isoflurane (1–1.5%). Once a surgical plane of anesthesia was confirmed (loss of pedal reflex), thrombi, adjacent vessel walls, and whole blood were collected. Death was confirmed by cessation of heartbeat following exsanguination.

### Establishment of the CLP model

The sepsis-induced ALI model was generated in both model and intervention groups using the CLP procedure, following Rittirsch et al. (2009) with minor modifications. Mice were anesthetized via inhalation of 2% isoflurane (RWD Life Science, China) and placed in a supine position. A 2-cm longitudinal incision was made slightly to the left of the midline, and the abdominal cavity was accessed through sequential layer separation. After exteriorizing the cecum, a 4-0 silk suture was used to ligate one-third of the cecum at a point approximately 1 cm from the distal tip. The cecum was then punctured once through both walls using a 22-gauge needle, and a small amount of fecal content was gently extruded before returning the cecum to the abdominal cavity (Li F. et al., 2024; Li X. et al., 2024). Sham-operated controls underwent cecal exposure without ligation or puncture. The abdominal wall was sutured in layers, and all animals received postoperative resuscitation with 1 mL pre-warmed (37 °C) sterile saline and routine supportive care, including warming and analgesia (Bao et al., 2023). Throughout the observation period from surgery to the predetermined experimental endpoints, no spontaneous deaths occurred in any group. Therefore, all surgically treated mice were included in the final analyses.

### FMT

To deplete the GM, recipient mice were administered a broad-spectrum antibiotic cocktail starting on day 7 post-modeling. The cocktail—comprising vancomycin (0.5 g/L), ampicillin (1 g/L), streptomycin (5 g/L), neomycin (1 g/L), and metronidazole (0.5 g/L) (all from Sigma-Aldrich, United States)—was dissolved in drinking water and administered continuously for 6 days. Twenty-four hours after antibiotic treatment, FMT was performed. To ensure viability and consistency of the transplanted microbiota, fresh fecal pellets were aseptically collected from healthy, syngeneic donor mice directly from the rectum and immediately processed under anaerobic conditions. Approximately 100 mg of fecal material (5–6 pellets) was suspended in 1 mL of sterile PBS and homogenized thoroughly in an anaerobic workstation. The homogenate was passed through a 20-μm nylon filter to remove large debris. Trypan blue staining confirmed that >90% of microorganisms in the resulting suspension were viable. The filtrate was administered to recipient mice by oral gavage (200 μL per mouse) within 15 min of preparation, with two doses given 3 days apart. Throughout the FMT procedure and during the pre-FMT antibiotic treatment, recipient mice continued to receive the same antibiotic cocktail in their drinking water for a total duration of 6 days. After transplantation, all recipient mice were housed individually in sterile isolators under gnotobiotic conditions for 8 weeks to ensure stable microbial colonization, followed by downstream experimental analyses (Li G. et al., 2023; Li Z. et al., 2023).

### Lentiviral vector construction and *in vivo* intervention

Specific pathogen-free (SPF)-grade male C57BL/6J mice were randomly assigned to three groups (*n* = 6 per group): FMT + sh-NC (negative control), FMT + sh-ITGAM (ITGAM knockdown), and FMT + sh-ITGB2 (ITGB2 knockdown). After verifying the knockdown efficiency of the lentiviral constructs, mice received tail-vein injections of the corresponding lentiviral suspensions (GeneChem, China) 3 days before surgery: sh-ITGAM targeting ITGAM, sh-ITGB2 targeting ITGB2, and sh-NC as a scrambled control. Each injection contained 200 μL of virus suspension (≥1 × 10^9^ TU/mL) at a dose of 4 × 10^8^ TU per mouse. All injections were performed in a biosafety cabinet, and mice were monitored for at least 30 min post-injection to ensure stability. Experimental procedures commenced 72 h after viral administration.

### Lung wet-to-dry weight ratio and bronchoalveolar lavage fluid cell count

At 48 h post-operation, mice were euthanized, and the left lung was lavaged three times with 1 mL of sterile PBS via tracheal cannulation. The resulting bronchoalveolar lavage fluid (BALF) was collected and centrifuged at 1500 rpm for 5 min. The cell pellet was resuspended in PBS, and total cell counts were determined using a hemocytometer. Simultaneously, the middle lobe of the right lung was excised and immediately weighed to determine wet weight. The tissue was then dried in an 80 °C oven for 48 h to obtain the dry weight. The lung wet-to-dry (W/D) weight ratio was calculated as an index of pulmonary edema.

### Hematoxylin and eosin staining

The left lung was fixed in 4% paraformaldehyde (P6148, Sigma-Aldrich, United States) for 24 h, dehydrated through graded ethanol, and embedded in paraffin. Tissue sections of 5 μm thickness were prepared using a rotary microtome (Leica RM2235). After deparaffinization and rehydration, the sections were stained with hematoxylin (H3136, Sigma-Aldrich, United States) for 5 min followed by eosin (E4009, Sigma-Aldrich, United States) for 2 min. Morphological analysis was conducted under a light microscope (Olympus BX43) at 400× magnification. Key histological parameters included pulmonary edema severity, hemorrhage extent, and infiltration of inflammatory cells.

### Evans Blue extravasation assay and pulmonary vascular permeability index

Pulmonary microvascular permeability was evaluated using the Evans Blue dye extravasation technique. Mice received an intravenous injection of Evans Blue (20 mg/kg; E2129, Sigma-Aldrich, United States) via the tail vein 30 min before sacrifice. After euthanasia, lungs were perfused with PBS for 10 min to remove intravascular dye. The left lung was excised, weighed, homogenized, and centrifuged, and absorbance of the supernatant was measured at 620 nm with correction at 740 nm using the formula: A620 (corrected) = A620 − (1.426 × A740 + 0.030). Evans Blue concentration was determined from a standard curve and expressed as μg/g wet lung weight. Pulmonary vascular permeability was further evaluated by calculating the ratio of total protein concentration in BALF to that in plasma, both quantified with a bicinchoninic acid (BCA) assay after centrifugation.

### Quantification of NET formation by MPO-DNA ELISA

The formation of NETs was assessed using an ELISA-based detection of MPO-DNA complexes. Briefly, 96-well plates were coated overnight at 4 °C with 75 μL/well of anti-MPO monoclonal antibody (5 μg/mL; 0400-0002, ABD Serotec, United Kingdom). After blocking with 1% BSA (125 μL/well), 40 μL of serum samples were added to each well, along with HRP-conjugated anti-DNA monoclonal antibody (11774425001, Roche, Switzerland). Plates were incubated on a shaker at 320 rpm for 2 h at ambient temperature, washed three times with PBS (200 μL/well), and developed using a chromogenic HRP substrate. Absorbance was measured at 405 nm using a Fluostar Optima microplate reader (BMG Labtech) after 40 min of incubation at 37 °C in the dark.

### Real-time quantitative polymerase chain reaction

Total RNA was extracted from tissue or cell samples using Trizol reagent (Invitrogen, 15596026). RNA concentration and purity were assessed via absorbance at 260/280 nm using a NanoDrop LITE spectrophotometer (ND-LITE-PR, Thermo Scientific, United States). Reverse transcription was performed with the PrimeScript RT reagent Kit with gDNA Eraser (RR047Q, TaKaRa, Japan), including a genomic DNA removal step prior to cDNA synthesis. Quantitative PCR was carried out with SYBR Green PCR Master Mix (4364344, Applied Biosystems, United States) on an ABI PRISM 7500 system. Primer sequences (Supplementary Table S2) were synthesized by TaKaRa, and the GAPDH gene was used as the internal control. The cycling conditions were 95 °C for 30 s, followed by 40 cycles of 95 °C for 5 s and 60 °C for 34 s. Each sample was run in technical triplicate.

Relative gene expression levels were calculated using the 2^−ΔΔCT^ method: ΔΔCT = (CT_target − CT_reference)_experimental − (Ct_target − Ct_reference)_control. Melting curve analysis confirmed the specificity of amplification, and all reactions included a no-template control (NTC) to ensure data integrity.

### Western blot

Tissue or cell samples were lysed in enhanced Radio-Immunoprecipitation Assay (RIPA) buffer containing protease inhibitors (P0013B, Beyotime, China). Lysates were centrifuged at 12,000 × g for 15 min at 4 °C, and the supernatants were collected. Protein concentrations were quantified using a BCA Protein Assay Kit (P0012, Beyotime, China) and adjusted uniformly to 2 μg/μL. Equal amounts of protein (30 μg per lane) were separated on 10% sodium dodecyl sulfate-polyacrylamide gel electrophoresis (SDS-PAGE) gels and transferred to polyvinylidene difluoride (PVDF) membranes (FFP39, Beyotime, China) using wet transfer. Membranes were blocked with 5% BSA (ST023, Beyotime, China) for 2 h at ambient temperature and incubated for 1 h with primary antibodies (Supplementary Table S3), all rabbit-derived, targeting ITGAM, ITGB2, β-catenin, ZO-1, and VE-cadherin. After three washes with TBST, membranes were incubated for 1 h with HRP-conjugated goat anti-rabbit secondary antibodies (ab6721, Abcam, United Kingdom; 1:2,000 dilution). Signals were visualized using the Pierce Enhanced Chemiluminescence (ECL) substrate (32209, Thermo Scientific, United States), applied evenly after mixing solutions A and B. Imaging was performed using a ChemiDoc MP system (Bio-Rad). β-actin served as the loading control, and band intensity was quantified using ImageJ software (v1.53). All experiments were independently replicated three times.

### Statistical analysis

All experiments were performed in triplicate, and data are presented as mean ± standard deviation (SD). Two-tailed *t*-tests were used for comparisons between two groups, while one-way ANOVA followed by Tukey’s *post-hoc* test was applied for multiple group comparisons. Statistical analyses were conducted using GraphPad Prism 9.0 (GraphPad Software, United States), and *p* < 0.05 was considered statistically significant.

## Results

### Dysbiosis of the GM exacerbates sepsis-induced ALI by compromising the pulmonary endothelial barrier

**Figure 1:**
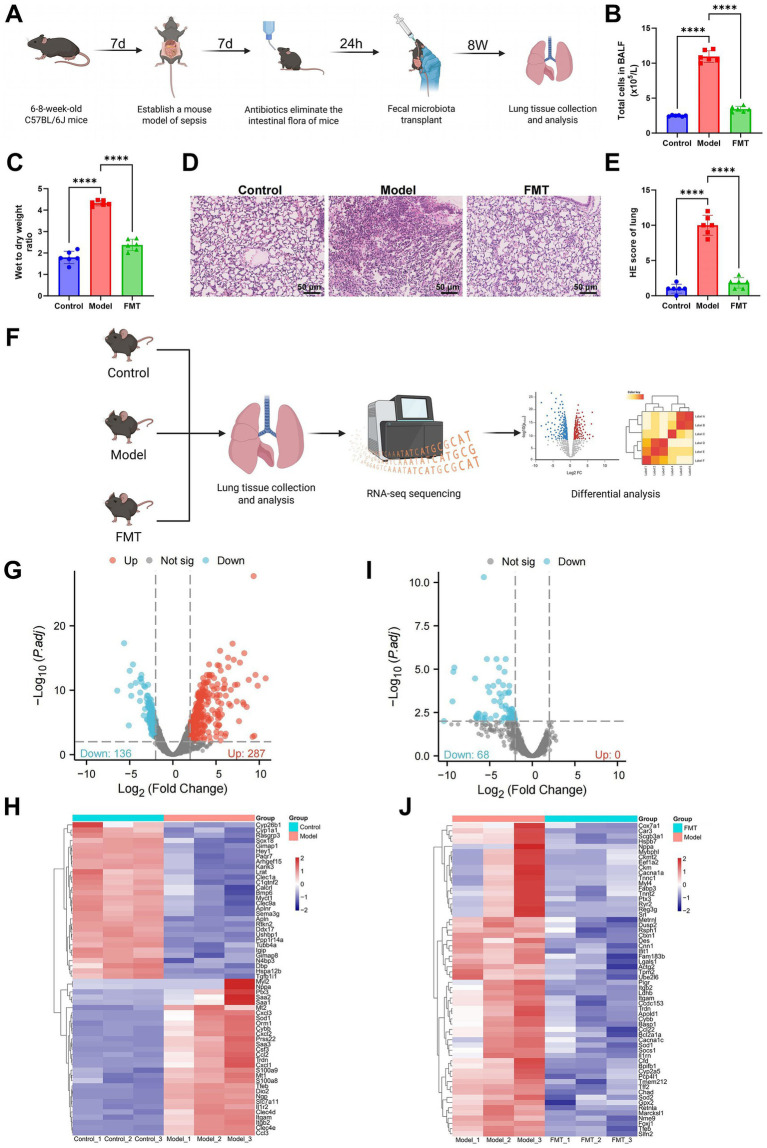
Transcriptomic analysis identifies key molecules involved in sepsis-induced ALI. **(A)** Schematic of the experimental workflow. The same cohort of mice first underwent CLP, followed by antibiotic treatment 7 days later. Twenty-four hours after antibiotic administration, mice received FMT, and lung tissues were subsequently collected for evaluation. **(B,C)** Quantification of total cells in BALF and measurement of lung W/D weight ratio. **(D,E)** H&E staining to evaluate lung histopathology (scale bar: 50 m). **(F)** Workflow of transcriptomic sequencing, illustrating tissue collection, RNA-seq, and differential expression analysis. **(G)** Volcano plot showing DEGs between the Model and Control groups. **(H)** Heatmap of gene expression profiles in the Model versus Control groups. **(I)** Volcano plot showing DEGs between the FMT and Model groups. **(J)** Heatmap of gene expression profiles in the FMT versus Model groups. Each group contained six mice. ^*^Indicates statistical significance between groups, ^****^p < 0.0001. For transcriptomic sequencing, three biological replicates were included per group (Control, Model, and FMT).

To investigate whether GM dysbiosis contributes to the development of sepsis-induced ALI, a murine CLP model was established (survival data in Supplementary Table S4), and FMT was applied as an intervention. We hypothesized that GM disruption may aggravate ALI by impairing the endothelial barrier, enhancing NET formation, and downregulating the expression of integrins ITGAM and ITGB2 (Figure 1A). Mice were randomly assigned to three groups (*n* = 6 per group): Control, CLP-induced Model, and FMT-treated. Lung injury and inflammation were assessed by quantifying total cells in BALF and calculating lung W/D weight ratios. The Model group exhibited markedly elevated BALF cellularity and W/D ratios relative to Controls, whereas both indices were significantly reduced following FMT (Figures 1B,C). Hematoxylin and eosin (H&E) staining further showed pronounced alveolar destruction, inflammatory infiltration, and interstitial edema in the Model group. These pathological abnormalities were substantially ameliorated in the FMT group, with partial restoration of alveolar structure (Figures 1D,E).

To elucidate the molecular mechanisms underlying the protective effects of FMT in sepsis-induced ALI, we performed high-throughput RNA sequencing on lung tissues from all three groups (Figure 1F). Differential expression analysis using the R package limma identified 423 DEGs between the Model and Control groups, implicating them in ALI pathogenesis (Figures 1G,H). Comparison between the FMT and Model groups yielded 68 DEGs, which are likely candidate targets modulated by FMT (Figures 1I,J).

**Figure 2:**
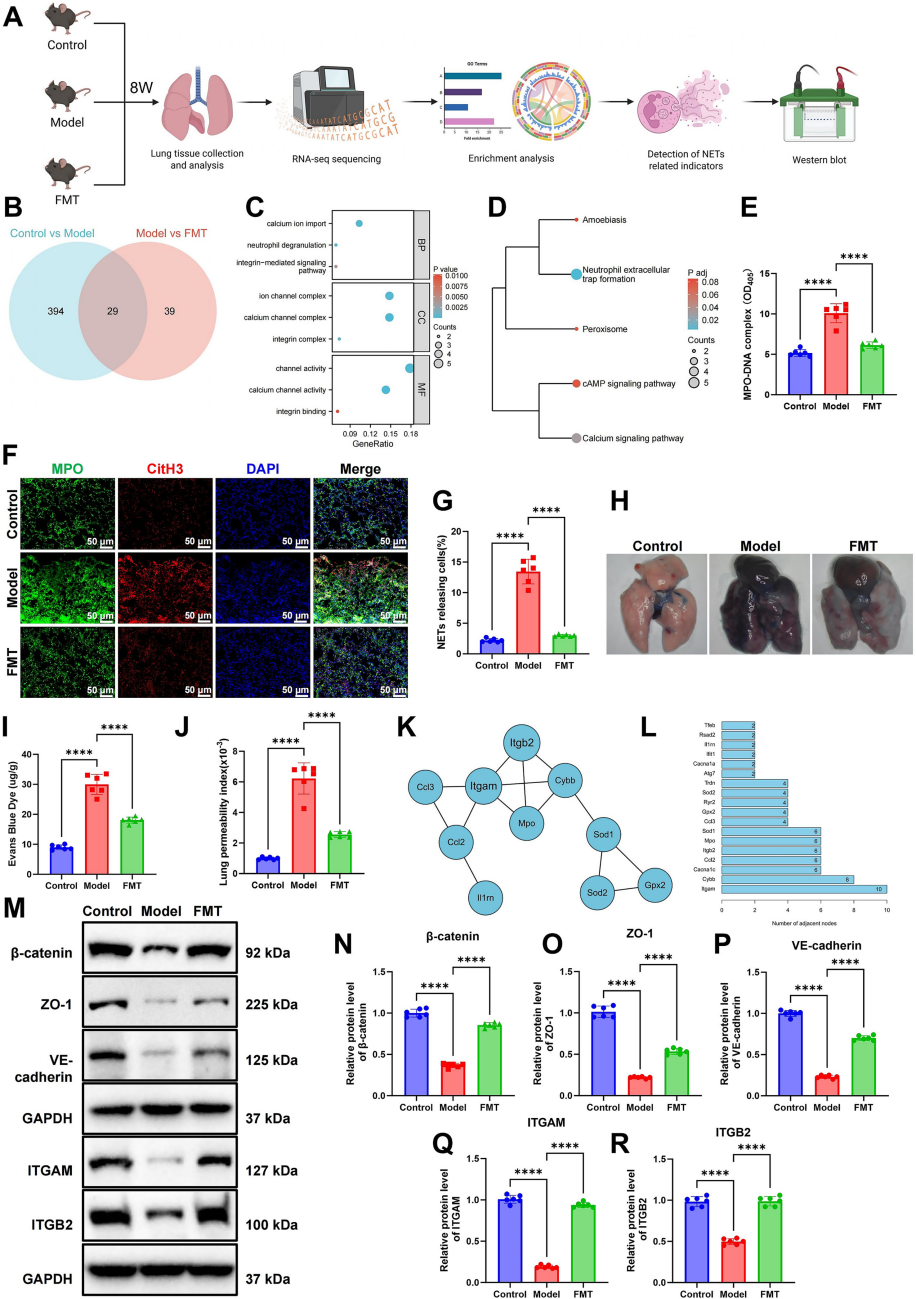
FMT attenuates NET activation and restores pulmonary endothelial barrier function. **(A)** Experimental workflow outlining transcriptomic differential gene enrichment analysis, NET quantification, and WB procedures for key protein detection. **(B)** Venn diagram showing the overlap of DEGs between Model vs. Control and FMT vs. Model groups. **(C)** GO BP enrichment analysis of DEGs, identifying pathways associated with GM-mediated protection against sepsis-induced ALI. **(D)** KEGG pathway enrichment of target genes involved in the pathogenesis of sepsis-related ALI. **(E)** ELISA quantification of MPO-DNA complexes in mouse plasma. **(F,G)** Immunofluorescence co-localization of CitH3 and MPO in lung tissue (scale bar = 50 μm). **(H,I)** Evans Blue dye leakage assay quantifying pulmonary vascular permeability. **(J)** Pulmonary permeability index calculated as the BALF protein/plasma protein ratio. **(K)** PPI network of overlapping DEGs constructed using the STRING database and visualized with Cytoscape. **(L)** Identification of central regulatory nodes in the PPI network based on degree centrality analysis. **(M–R)** WB analysis (M) and statistics of β-catenin **(N)**, ZO-1 **(O)**, VE-cadherin **(P)**, ITGAM **(Q)**, and ITGB2 **(R)** expression in lung tissues. Each group included six mice. ^*^p < 0.05, ^***^p < 0.001, and ^****^p < 0.0001 indicate statistical significance between groups. Transcriptomic sequencing was performed on three biological replicates per group (Control, Model, and FMT).

Intersection analysis of DEGs from both comparisons was conducted to identify potential key regulators of FMT-mediated lung protection (Figures 2A,B). GO and KEGG enrichment analyses showed that intersecting DEGs were significantly enriched in BP such as calcium ion import, neutrophil degranulation, and integrin-mediated signaling (Figure 2C). KEGG analysis further indicated upregulation of pathways associated with NET formation (Figure 2D).

To validate the transcriptomic findings and further examine the impact of GM on NET formation during sepsis-induced ALI, plasma levels of MPO-DNA complexes were quantified by ELISA. Compared to Controls, the Model group showed a significant increase in MPO-DNA levels, which was markedly reduced in the FMT group (Figure 2E). Immunofluorescence staining of lung tissue confirmed these findings, demonstrating strong CitH3 and MPO co-localization signals in the Model group, indicative of enhanced NET formation, which were attenuated by FMT (Figures 2F,G). These results suggest that FMT effectively suppresses NET formation and alleviates inflammatory injury. We next assessed pulmonary vascular permeability using Evans Blue dye extravasation and calculated the vascular permeability index. The Model group exhibited significantly elevated dye accumulation and permeability indices, indicating severe endothelial dysfunction, both of which were reduced following FMT (Figures 2H–J).

Subsequently, a PPI network was constructed using the STRING database, and key genes were identified based on degree centrality (Figure 2K). The results revealed that ITGAM exhibited the highest interaction score, and both ITGAM and ITGB2 occupied central nodes within the network, suggesting their potential involvement in direct or indirect regulatory interactions (Figure 2L). Consistent with these findings, western blot (WB) analysis showed pronounced downregulation of the endothelial tight junction proteins β-catenin, ZO-1, and VE-cadherin, as well as the integrin subunits ITGAM and ITGB2, in the Model group. Notably, FMT largely restored the expression of all these proteins to near-baseline levels (Figures 2M–R).

Collectively, these findings indicate that dysregulation of the GM may facilitate the activation of NETs, while the expression of integrins ITGAM and ITGB2 is closely linked to the progression of sepsis-induced ALI. Our data suggest that microbiota imbalance exacerbates pulmonary endothelial barrier disruption by promoting NET formation and suppressing integrin expression, whereas FMT exerts a protective effect by partially reversing these pathological alterations.

### The role of GM-derived NETs in sepsis-induced ALI

**Figure 3:**
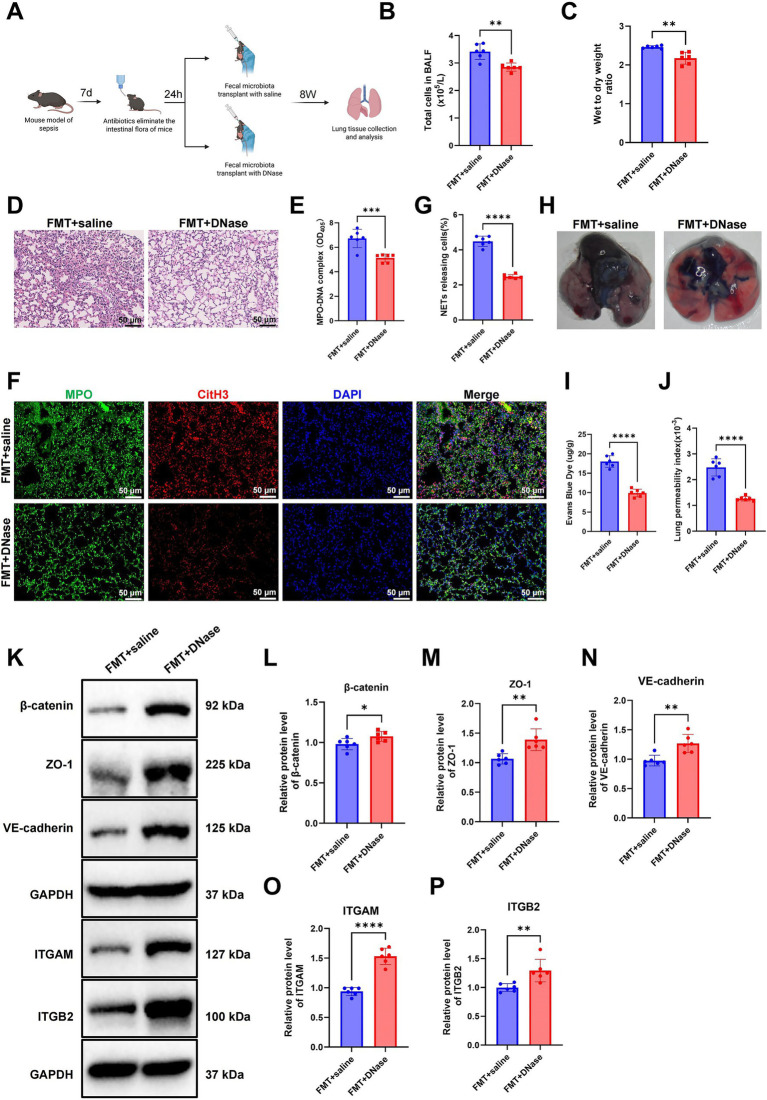
DNase I attenuates pulmonary inflammation and barrier disruption by inhibiting NETs. **(A)** Schematic of the experimental workflow. The same cohort of mice first underwent CLP, followed by antibiotic treatment 7 days later. Twenty-four hours after antibiotic administration, mice received FMT, and lung tissues were subsequently collected for evaluation. **(B,C)** Quantification of total cells in BALF and measurement of lung W/D weight ratio. **(D)** Representative H&E-stained lung sections illustrating histopathological changes (scale bar: 50 μm). **(E)** ELISA quantification of MPO-DNA complex levels in plasma. **(F,G)** Immunofluorescence staining showing co-localization of CitH3 and MPO in lung tissues (scale bar: 50 μm). **(H,I)** Evans Blue dye extravasation assay evaluating pulmonary vascular leakage. **(J)** Calculation of pulmonary permeability index (BALF protein/plasma protein ratio). **(K–P)** WB analysis **(K)** and statistics of β-catenin **(L)**, ZO-1 **(M)**, VE-cadherin **(N)**, ITGAM **(O)**, and ITGB2 **(P)** expression in lung tissue. Each group included six mice. ^*^p < 0.05, ^**^p < 0.01, ^***^p < 0.001, and ^****^p < 0.0001. Statistical significance was determined by pairwise comparison.

To investigate the contribution of NETs in GM-mediated sepsis-induced ALI, we combined DNase I treatment—with the purpose of degrading the DNA backbone of NETs—with FMT to examine its protective effects on lung tissue (Figure 3A). Mice were assigned to two groups: FMT plus saline and FMT plus DNase I. BALF analysis showed a significant reduction in total cell counts in the FMT + DNase group relative to the FMT + saline group, indicating diminished inflammatory cell infiltration. Concurrently, the lung W/D weight ratio was markedly decreased, suggesting reduced pulmonary edema (Figures 3B,C). H&E staining further supported these findings, showing pronounced alveolar wall thickening, interstitial edema, and neutrophil accumulation in the FMT + saline group, which were notably alleviated in the FMT + DNase group (Figure 3D).

To confirm the ability of DNase I to degrade the DNA backbone of NETs, plasma levels of MPO-DNA complexes were quantified by ELISA. Results demonstrated a significant decrease in MPO-DNA levels in the FMT + DNase group relative to controls (Figure 3E). Immunofluorescence staining corroborated these findings, showing enhanced co-localization of citrullinated histone H3 (CitH3) and MPO in the FMT + saline group, which was markedly diminished following DNase I treatment (Figures 3F,G). These results indicate that DNase I effectively degrades the DNA scaffold of NETs, thereby alleviating NET-driven inflammatory responses.

To further evaluate the role of NETs in pulmonary barrier integrity, Evans Blue dye extravasation assays revealed significantly reduced vascular leakage in the FMT + DNase group compared to the FMT + saline group (Figures 3H,I). The pulmonary permeability index, calculated as the BALF-to-plasma total protein ratio, was significantly reduced in the DNase-treated group, further indicating improved endothelial barrier function (Figure 3J). WB analysis showed marked upregulation of the integrin subunits ITGAM and ITGB2 in lung tissues from the FMT + DNase group compared with the FMT + saline group. In addition, DNase I treatment substantially restored the expression of the tight junction proteins β-catenin, ZO-1, and VE-cadherin (Figures 3K–P).

Collectively, these findings indicate that NETs play a critical role in GM—mediated septic lung injury. The underlying mechanism involves dysregulated integrin expression and disruption of endothelial tight junction structures, leading to impaired pulmonary endothelial barrier integrity. These results suggest that targeting NETs—through the degradation of their DNA scaffold—may mitigate endothelial damage, preserve the protective endothelial glycocalyx, and hold therapeutic potential for sepsis-associated ALI.

### NETs induce inflammatory activation and barrier dysfunction in pulmonary endothelial cells

**Figure 4:**
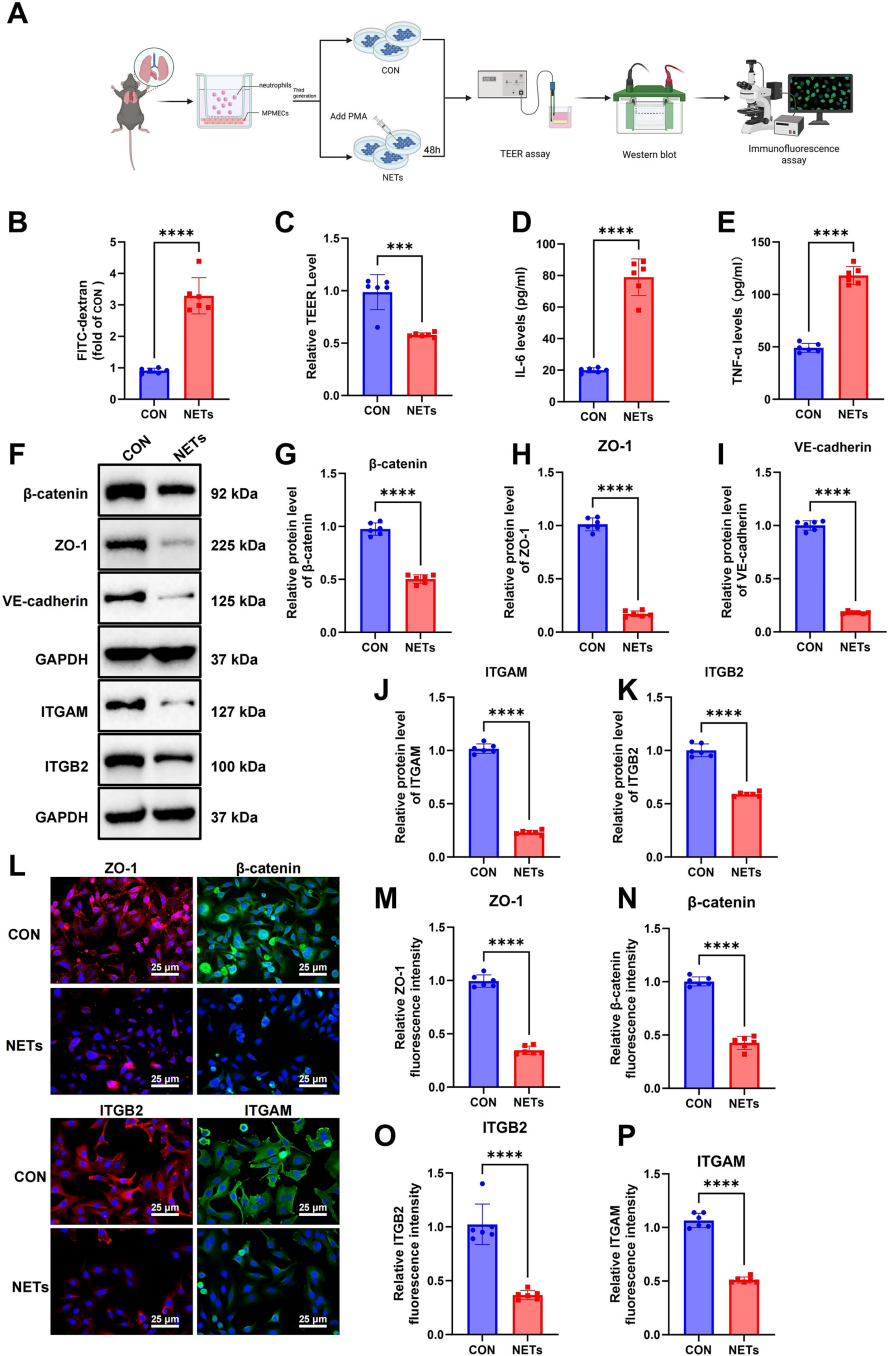
NETs impair pulmonary endothelial barrier structure and function. **(A)** Schematic overview of the experimental workflow, including NET generation, treatment of MPMECs, and subsequent assays. **(B)** FITC-dextran permeability assay assessing monolayer permeability of MPMECs. **(C)** TEER measurements evaluating endothelial barrier integrity. **(D,E)** ELISA quantification of IL-6 and TNF-α levels in MPMEC culture supernatants. **(F–K)** WB analysis **(F)** and statistics of β-catenin **(G)**, ZO-1 **(H)**, VE-cadherin **(I)**, ITGAM **(J)**, and ITGB2 **(K)** protein expression. **(L–P)** Immunofluorescence staining of β-catenin and ZO-1 localization, as well as ITGAM and ITGB2 expression. Scale bar: 25 μm. Cell experiments were repeated six times. ^*^Indicates significant difference between groups; ^***^p < 0.001 and ^****^p < 0.0001.

To investigate the impact of NETs on the barrier integrity of MPMECs, we isolated primary MPMECs and neutrophils from mice and established an *in vitro* co-culture system comprising a control group (CON) and a NET-treated group. All experiments were independently repeated three times (Figure 4A). In the FITC-dextran permeability assay, the NET-treated group exhibited a significantly increased permeability compared to the CON group, indicating compromised endothelial barrier function (Figure 4B). Consistently, TEER measurements revealed a marked reduction in resistance following NET exposure, further reflecting impaired barrier integrity (Figure 4C). Together, these findings demonstrate that NETs disrupt the monolayer barrier function of pulmonary endothelial cells.

To evaluate the proinflammatory effects of NETs, cytokine levels in culture supernatants were measured by ELISA. The NET group exhibited markedly increased IL-6 and TNF-α secretion compared with the CON group, indicating strong proinflammatory activation of endothelial cells following NET exposure (Figures 4D,E). WB analysis further revealed a pronounced downregulation of the integrins ITGAM and ITGB2, as well as tight junction proteins including β-catenin, ZO-1, and VE-cadherin in the NET group (Figures 4F–K), indicating that NETs not only suppress integrin expression but also compromise cell–cell junction integrity.

Immunofluorescence staining provided additional insight into the structural effects of NETs on endothelial cells. Compared with the CON group, the NET-treated cells exhibited disrupted localization of β-catenin and ZO-1, along with clear disintegration of intercellular junctions. Moreover, the membrane-associated expression of ITGAM and ITGB2 was markedly reduced (Figures 4L–P).

These observations corroborate the biochemical findings, confirming that NETs impair endothelial barrier function by disrupting the localization of integrins and junctional proteins, thereby undermining the structural stability of pulmonary endothelial cells.

### NETs downregulate ITGAM and ITGB2, disrupting pulmonary endothelial barrier integrity

**Figure 5:**
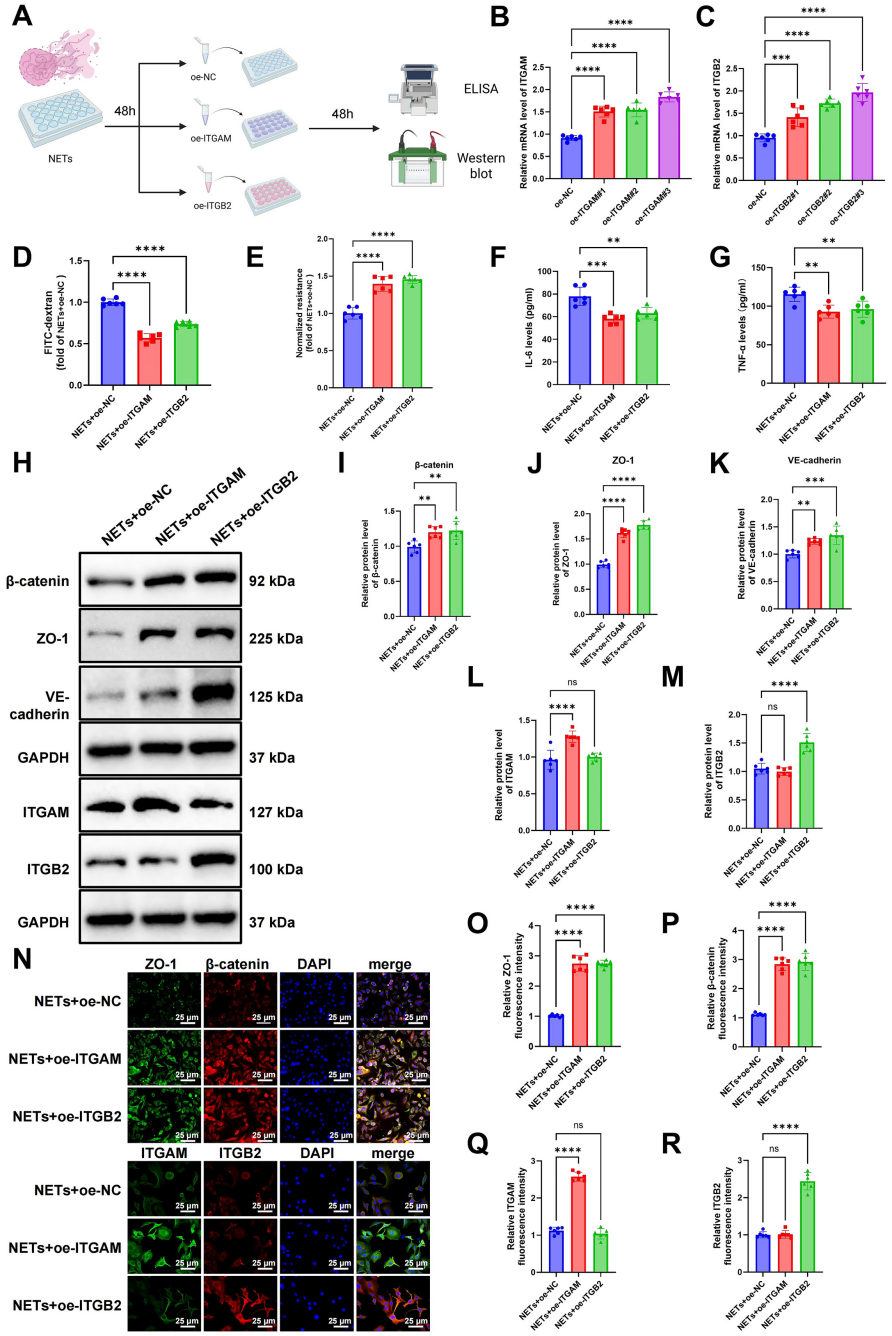
Protective effects of ITGAM and ITGB2 overexpression on NET-induced pulmonary endothelial injury. **(A)** Schematic diagram of the experimental workflow: neutrophil-induced NET generation, MPMEC infection with ITGAM or ITGB2 lentivirus, and subsequent functional assays. **(B,C)** RT-qPCR analysis confirming overexpression efficiency of ITGAM and ITGB2. **(D)** FITC-dextran permeability assay assessing MPMEC barrier integrity. **(E)** TEER measurement of MPMEC monolayer integrity. **(F,G)** ELISA quantification of IL-6 and TNF-α levels in MPMEC culture supernatants. **(H–M)** WB analysis **(H)** and statistics of β-catenin **(I)**, ZO-1 **(J)**, VE-cadherin **(K)**, ITGAM **(L)**, and ITGB2 **(M)** expression in MPMECs. **(N–R)** Immunofluorescence imaging of β-catenin and ZO-1 localization, and ITGAM and ITGB2 expression in MPMECs. Scale bar: 25 μm. All cell experiments were independently repeated six times. ns indicates no significant difference; ^*^p < 0.05, ^**^p < 0.01, ^***^p < 0.001, and ^****^p < 0.0001.

To elucidate the roles of ITGAM and ITGB2 in NET-induced pulmonary endothelial barrier dysfunction, we established three groups of MPMEC models—NET + oe-NC, NET + oe-ITGAM, and NET + oe-ITGB2—via lentiviral-mediated overexpression of ITGAM or ITGB2 following NET exposure. Each experiment was independently replicated three times (Figure 5A). Real-time quantitative polymerase chain reaction (RT-qPCR) confirmed efficient overexpression of both genes (Figures 5B,C). FITC-dextran permeability assays revealed that cells in the NET + oe-ITGAM and NET + oe-ITGB2 groups exhibited significantly reduced permeability compared to the NET + oe-NC group, indicating that upregulation of integrins markedly restored endothelial barrier function (Figure 5D). Consistent with this, TEER measurements demonstrated a notable decline in barrier integrity in the NET + oe-NC group, whereas TEER values were significantly elevated in the NET + oe-ITGAM and NET + oe-ITGB2 groups (Figure 5E), suggesting effective recovery of barrier function upon integrin overexpression.

ELISA results revealed that the NET + oe-ITGAM and NET + oe-ITGB2 groups exhibited markedly reduced IL-6 and TNF-α secretion compared with the NET + oe-NC group (Figures 5F,G), implying a mitigating effect of integrin upregulation on NET-induced inflammatory responses. WB analyses further demonstrated increased expression of ITGAM and ITGB2 proteins in their respective overexpression groups. Concurrently, tight junction-associated proteins—including β-catenin, ZO-1, and VE-cadherin—were significantly restored compared to the control group (Figures 5H–M), underscoring a close association between integrin expression and tight junction integrity.

Immunofluorescence staining revealed disrupted localization and discontinuous distribution of β-catenin and ZO-1 in the NET + oe-NC group, along with apparent intercellular gaps. In contrast, these proteins were relocalized to the cell borders in the NET + oe-ITGAM and NET + oe-ITGB2 groups, accompanied by enhanced membrane-associated fluorescence of ITGAM and ITGB2 (Figures 5N–R).

Collectively, these findings indicate that ITGAM and ITGB2 not only modulate NET-induced cellular adhesion dynamics but also play pivotal roles in preserving endothelial barrier architecture.

### Disruption of the GM exacerbates sepsis-induced ALI via NET-mediated downregulation of ITGAM and ITGB2

**Figure 6:**
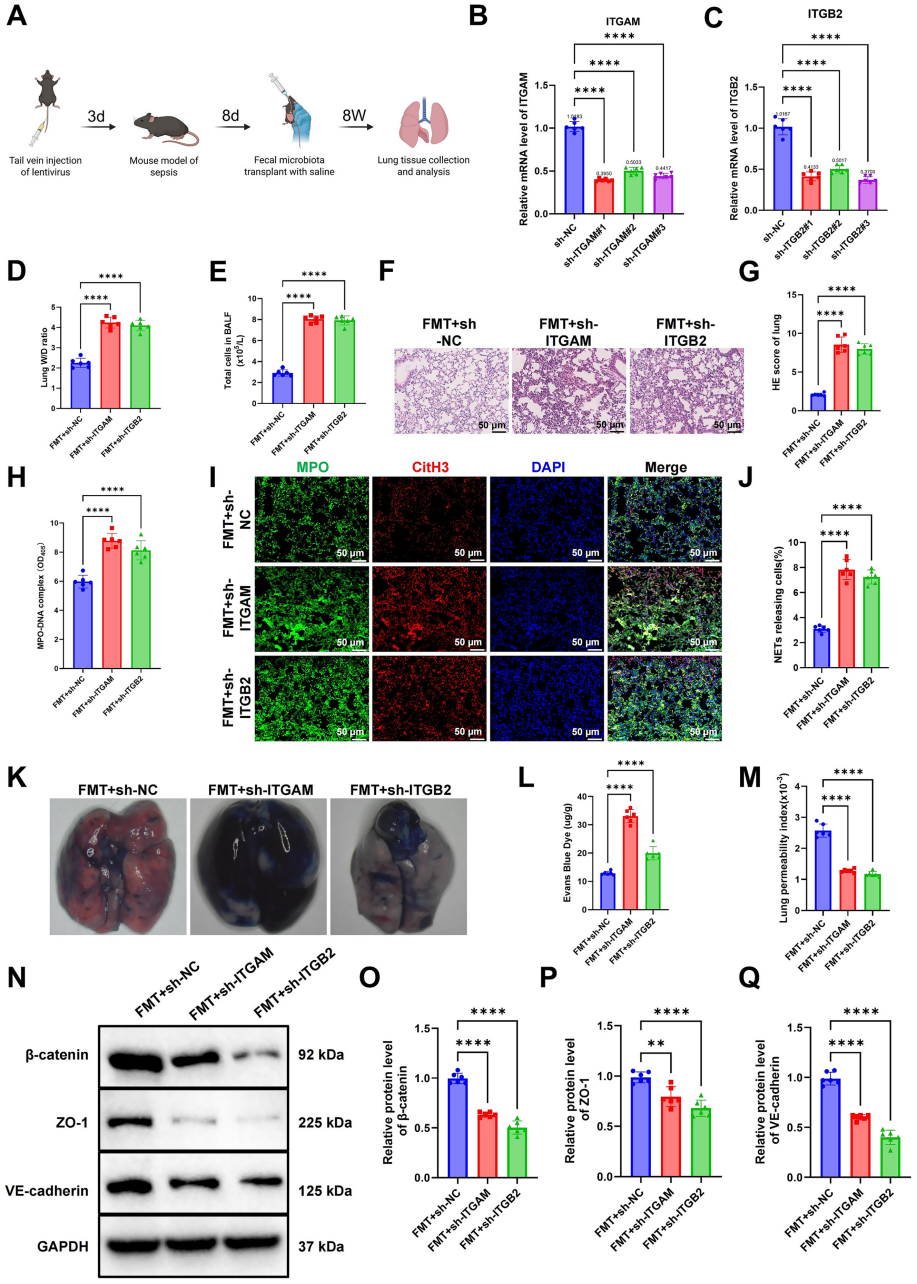
Silencing *ITGAM* or *ITGB2* exacerbates ALI and disrupts the endothelial barrier. **(A)** Schematic illustration of the experimental workflow showing *ITGAM* or *ITGB2* knockdown following FMT in mice. **(B,C)** RT-qPCR analysis confirming the efficiency of *ITGAM* and *ITGB2* knockdown by lentiviral transduction. **(D,E)** Quantification of total cell counts in BALF and lung tissue W/D weight ratios. **(F,G)** H&E staining of lung sections showing structural damage and inflammatory infiltration (scale bar = 50 μm). **(H)** ELISA quantification of the NET marker MPO-DNA complex in plasma. **(I,J)** Immunofluorescence visualization of NET distribution in lung tissues (scale bar = 50 μm). **(K,L)** Evans Blue dye leakage assay to assess pulmonary vascular permeability. **(M)** Calculation of lung permeability index (BALF protein/plasma protein ratio). **(N–Q)** WB analysis **(N)** and statistics of β-catenin **(O)**, ZO-1 **(P)**, and VE-cadherin **(Q)** protein expression in lung tissues. Each group included six mice. ^**^p < 0.01 and ^****^p < 0.0001 for group comparisons.

To further elucidate the critical role of ITGAM and ITGB2 in NET-mediated ALI, a murine model with targeted knockdown of either ITGAM or ITGB2 was established on the basis of FMT. Mice were divided into three groups: FMT + sh-NC, FMT + sh-ITGAM, and FMT + sh-ITGB2 (Figure 6A). RT-qPCR analysis confirmed the silencing efficiency of lentiviral constructs, and the shRNA sequences with the strongest knockdown efficiency—sh-ITGAM#1 and sh-ITGB2#3—were selected for subsequent experiments (Figures 6B,C). Compared with the FMT + sh-NC group, mice in both knockdown groups exhibited marked deterioration in lung function, evidenced by significantly elevated total cell counts in BALF and increased lung W/D weight ratios, indicating exacerbated pulmonary edema (Figures 6D,E). Histological analysis (H&E staining) further revealed extensive alveolar structural disruption and heightened inflammatory cell infiltration in both the sh-ITGAM and sh-ITGB2 groups (Figures 6F,G).

ELISA results demonstrated a substantial increase in plasma MPO-DNA complexes in the knockdown groups relative to controls, consistent with enhanced NET formation. Immunofluorescence staining corroborated these findings, showing increased NET accumulation in lung tissues (Figures 6H–J), suggesting that suppression of ITGAM or ITGB2 may impair NET clearance or augment their formation, thereby intensifying inflammation.

Evans Blue dye extravasation assays revealed significantly increased vascular leakage and lung permeability indices in the knockdown groups compared to FMT + sh-NC mice (Figures 6K–M). WB analyses showed a pronounced reduction in key endothelial junction proteins, including β-catenin, ZO-1, and VE-cadherin, further indicating compromised endothelial barrier integrity (Figures 6N–Q).

Collectively, these findings indicate that sepsis induces robust NET formation, while the GM modulates the expression of ITGAM and ITGB2, thereby preserving endothelial structure and barrier integrity in the presence of NETs.

## Discussion

The GM, often referred to as a “hidden organ” due to its pivotal role in modulating host immune homeostasis and barrier integrity, has garnered increasing attention in the context of sepsis and multiple organ dysfunction syndrome (Yoo et al., 2020; Barbara et al., 2021). Prior research has primarily examined the immunomodulatory roles of gut microbiota—derived metabolites, such as SCFAs and bile acids, particularly in regulating Treg differentiation and macrophage activation (Gasaly et al., 2021; Li G. et al., 2023; Li Z. et al., 2023). Multiple omics platforms, including metagenomics, metabolomics, proteomics, and transcriptomics, have been widely applied to characterize the interactions among microbial composition, environmental factors, and mucosal immune responses in inflammatory bowel disease (IBD). In addition, numerous bile acid-derived metabolites have been identified as key regulators of intestinal immunity, offering important insights into how bile acids shape the pathogenesis and progression of IBD and colorectal cancer (CRC) (Cai et al., 2022). However, the contribution of the GM to distal organ injury via neutrophil activation remains underexplored, especially within the domain of sepsis-induced ALI (Ma et al., 2020). Previous evidence has shown that inhibiting NETs, either as a standalone treatment or as an adjuvant to antibiotics, can improve survival in the CLP model and alter the GM, thereby exerting important protective effects during sepsis (de Araujo et al., 2023). In this study, using a CLP murine model of sepsis combined with FMT, we demonstrate that dysbiosis markedly promotes the formation of NETs, exacerbating pulmonary inflammation and barrier disruption. These findings unveil a novel mechanistic link within the gut-lung axis, advancing our molecular understanding of long-distance microbial-immune crosstalk in pulmonary pathophysiology.

NETs, once primarily recognized for their antimicrobial functions as components of the innate immune response, have increasingly been implicated in the pathogenesis of infectious diseases, malignancies, and autoimmune disorders (Hawez et al., 2021; De Meo and Spicer, 2021). Emerging evidence demonstrates that during systemic inflammatory conditions such as sepsis, neutrophils become excessively activated, releasing large quantities of NETs that contribute to microvascular obstruction, tissue injury, and the propagation of inflammation—particularly within the lungs, an organ highly susceptible due to its dense vascular network (Scozzi et al., 2022; Ding et al., 2022). In both *in vivo* and *in vitro* experiments, our study observed a marked increase in NETs correlated with impaired pulmonary endothelial barrier function, reduced expression of tight junction proteins, and heightened secretion of pro-inflammatory cytokines. Notably, and in contrast to previous findings (Zhu et al., 2024), our work further demonstrates—through NET induction assays—the direct damaging effect of NETs on endothelial barrier integrity. By integrating these findings with the observed alterations in integrin expression, we propose a mechanistic framework in which NETs disrupt endothelial anchorage to the basement membrane. The basement membrane, enriched in laminins and collagen IV, anchors endothelial cells via integrin-containing FA complexes (Pulous and Petrich, 2019). These adhesion complexes interact dynamically with the cytoskeleton under mechanical stress and thus play essential roles in endothelial mechanotransduction (Okamoto et al., 2021). Based on this evidence, our study is the first to suggest that NETs may compromise the pulmonary endothelial barrier by modulating cell adhesion molecules, providing a more refined mechanistic explanation for NET-mediated endothelial injury.

Integrins ITGAM (CD11b) and ITGB2 (CD18), which form the β2 integrin complex, are well-established mediators of leukocyte adhesion, transendothelial migration, and pathogen clearance (Faridi et al., 2013). While their roles in immune regulation and infection have been described, their specific involvement in pulmonary endothelial barrier maintenance remains insufficiently characterized (Lin et al., 2023). Through transcriptomic profiling and functional assays, we found that ITGAM and ITGB2 are significantly downregulated in the lungs of septic mice, with expression levels positively correlating with barrier function. Moreover, enforced expression of either integrin *in vitro* effectively reversed NET-induced endothelial injury, restoring tight junction protein expression and suppressing inflammatory cytokine production. These results suggest that, beyond their established functions in neutrophils, ITGAM and ITGB2 may serve as key regulators of endothelial homeostasis. Our findings broaden the functional repertoire of the integrin family and highlight their therapeutic potential in preserving lung microvascular integrity during septic injury.

Although our study primarily focused on the downstream NET-integrin-endothelial axis, it is important to acknowledge that we did not directly perform 16S rRNA sequencing or metabolomic profiling within this experimental framework. Nevertheless, extensive literature has established that CLP-induced sepsis consistently leads to reduced microbial diversity, decreased *Firmicutes*, expansion of *Proteobacteria*, and loss of beneficial genera such as *Lactobacillus*, *Bifidobacterium*, and *Ruminococcus* (Sun et al., 2022; Lou et al., 2023; Barlow et al., 2022). FMT has also been validated across multiple independent studies to restore microbial balance in CLP models. Based on this well-established evidence, we employed FMT as a reliable microbiota-targeted intervention, and its functional efficacy was reflected by downstream immunological and endothelial improvements.

Furthermore, microbially derived metabolites, particularly SCFAs such as butyrate, acetate, and propionate, have been shown to suppress NET formation, modulate neutrophil activation, and maintain endothelial barrier stability via GPR41/GPR43 signaling pathways (Wang et al., 2017; Mann et al., 2024). Although metabolomic assays were not performed here, integrating these mechanistic findings provides additional context for the immunomodulatory potential of FMT. Future work combining metagenomics, metabolomics, and host transcriptomics will help identify the specific microbial taxa and metabolites that regulate the NET-integrin axis.

Methodologically, this study integrates multi-omics approaches and systems biology frameworks, anchored in high-throughput transcriptomic sequencing. By combining GO, KEGG pathway enrichment, and PPI network analyses, we identified core regulatory pathways and pivotal genes from a systems-level perspective, overcoming the limitations of traditional gene selection based on empirical assumptions. Notably, PPI analysis highlighted ITGAM and ITGB2 as central nodes in immune signaling and barrier integrity, providing a robust theoretical basis for subsequent functional validation. In contrast to conventional studies that focus narrowly on isolated biochemical pathways (Zhao et al., 2023; Shen et al., 2006), our approach merges large-scale data mining with *in vivo* and *in vitro* validation, constructing a coherent mechanistic chain from transcriptomic shifts to phenotypic manifestations. This strategy presents a scalable model for dissecting multifactorial disease mechanisms and may inform future studies of similarly complex pathologies.

Importantly, FMT not only markedly reversed the ALI phenotype induced by dysbiosis but also significantly suppressed NET formation and restored integrin expression at the molecular level. These findings affirm the modulatory capacity of the gut-lung axis from both functional and mechanistic standpoints. While FMT has been primarily investigated in the context of intestinal disorders and *Clostridioides difficile* infections (Patwa et al., 2022; Chen et al., 2024), this study pioneers its application in mitigating remote organ injury in sepsis and demonstrates promising therapeutic outcomes. This suggests that microbiota-targeted interventions may transcend conventional organ-specific treatments and evolve into systemic therapeutic strategies. These results not only broaden the clinical relevance of FMT but also lay the groundwork for exploring other microbiota-based interventions, such as probiotics and microbial metabolite therapies.

Based on these findings, we propose an original regulatory model—the “gut microbiota-NET-integrin axis”—which systematically delineates how microbial dysbiosis can influence distal pulmonary endothelial integrity through immune modulation. This model integrates key processes including microbial shifts, NET induction, integrin downregulation, and endothelial barrier disruption, thereby filling a significant gap in our understanding of sepsis-induced ALI. It further underscores the value of interdisciplinary, multi-tiered research in unraveling complex immune-barrier dynamics. Critically, this framework offers a mechanistic rationale for the development of NET inhibitors or integrin agonists as targeted therapies, while highlighting the translational potential of microbiota-based modulation in systemic inflammatory diseases.

Despite the series of findings demonstrated using the CLP mouse model, several limitations of this study should be acknowledged. First, the work relied on a single murine sepsis model using male C57BL/6J mice. Although this model serves as an effective tool for proof-of-concept studies, inherent differences in pathophysiology, immune responses, and microbiota composition between mice and humans remain. The immunological background and genetic characteristics of this specific strain may not fully represent the diversity observed across other mouse strains or human populations. Second, all experiments were conducted under SPF conditions and involved the use of a potent antibiotic cocktail to deplete endogenous microbiota. Such highly standardized environmental conditions and strong microbial perturbation differ substantially from the heterogeneous microbiota backgrounds and medication histories present in clinical patients. Finally, the CLP model induces an acute and diffuse peritonitis, which may not fully recapitulate the disease course or complexity of all forms of sepsis encountered in clinical settings. To bridge this translational gap, future research strategies should include the use of humanized mouse models or *in vitro* co-culture systems incorporating patient-derived neutrophils and endothelial cells. Additionally, assessing NET-related markers, integrin expression, and endothelial function in patient blood and bronchoalveolar lavage fluid will be valuable for advancing NETs as a potential therapeutic target toward clinical application.

In conclusion, our study reveals a novel mechanism whereby gut dysbiosis exacerbates sepsis-induced ALI by promoting NET formation and downregulating ITGAM and ITGB2, leading to compromised pulmonary endothelial barrier function. These findings not only enrich the molecular framework of the gut-lung axis but also introduce a new conceptual pathway—the “NET-integrin-endothelial barrier” cascade—with significant implications for both basic research and clinical translation. Clinically, ITGAM and ITGB2 may serve as candidate biomarkers for assessing lung barrier integrity, while targeted modulation of the GM or NET/integrin pathways may represent next-generation therapeutic strategies. Nonetheless, limitations remain, including the translational gap between animal models and human sepsis, as well as the need for precise delineation of the signaling crosstalk between GM and NETs. Future studies incorporating single-cell sequencing, integrative multi-omics, and patient-derived samples will be essential for refining these mechanisms and advancing personalized therapeutic approaches for sepsis-induced ALI.

## Conclusion

This study systematically demonstrates that GM dysbiosis promotes the formation of NETs, leading to a significant downregulation of the integrins ITGAM and ITGB2 in pulmonary tissue. This molecular alteration compromises the integrity of the pulmonary endothelial barrier, resulting in increased vascular permeability and exacerbating sepsis-induced ALI. Overexpression of ITGAM or ITGB2, both *in vitro* and *in vivo*, was found to effectively restore endothelial barrier function, underscoring their protective roles.

By adopting a “gut-lung axis” perspective, this work elucidates a specific immunological pathway through which microbial perturbations contribute to ALI, offering a novel mechanistic framework for understanding the pathogenesis of sepsis-induced ALI. These findings identify ITGAM and ITGB2 as promising therapeutic targets. Moreover, the clinical potential of FMT and NET inhibition is highlighted, suggesting their utility in the context of precision immunotherapy for severe infections.

However, the generalizability of these mechanisms to human physiology remains unconfirmed, and the specific pathogenic taxa or microbial metabolites involved have yet to be characterized. Future studies integrating metagenomics and single-cell transcriptomics may provide deeper insights into the dynamic interplay between the microbiome, immune responses, and barrier function. Additionally, the development of ITGAM/ITGB2-targeted nanodelivery systems could facilitate the translation of these mechanistic insights into individualized therapeutic strategies.

## Data Availability

The original contributions presented in the study are included in the article/Supplementary material, further inquiries can be directed to the corresponding author.

## Footnotes

1
https://www.xiantaozi.com/

2
https://string-db.org/

3
http://www.ncbi.nlm.nih.gov

## Glossary

ANOVA: Analysis of variance
ALI: Acute lung injury
BALF: Bronchoalveolar lavage fluid
BCA: Bicinchoninic acid
BP: Biological process
BSA: Bovine serum albumin
CC: Cellular component
CLP: Cecal ligation and puncture
DAPI: 4′,6-Diamidino-2-phenylindole
DEGs: Differentially expressed genes
ECL: Enhanced chemiluminescence
ELISA: Enzyme-linked immunosorbent assay
FBS: Fetal bovine serum
FITC: Fluorescein isothiocyanate
FMT: Fecal microbiota transplantation
GO: Gene Ontology
H&E: Hematoxylin and eosin
HRP: Horseradish peroxidase
ITGAM: Integrin alpha M
ITGB2: Integrin beta 2
KEGG: Kyoto Encyclopedia of Genes and Genomes
MF: Molecular function
MPMECs: Mouse pulmonary microvascular endothelial cells
MPO: Myeloperoxidase
NETs: Neutrophil extracellular traps
PBS: Phosphate-buffered saline
PMA: Phorbol 12-myristate 13-acetate
PPI: Protein–protein interaction
PVDF: Polyvinylidene difluoride
RIPA: Radio-immunoprecipitation assay
RT-qPCR: Real-time quantitative polymerase chain reaction
SDS-PAGE: Sodium dodecyl sulfate-polyacrylamide gel electrophoresis
SPF: Specific pathogen-free
TEER: Transendothelial electrical resistance
TNF-α: Tumor necrosis factor-alpha
WB: Western blot
W/D: Wet-to-dry
