# Supplementary material for: Gut microbiota dysbiosis aggravates sepsis-induced lung injury by promoting neutrophil extracellular traps and suppressing host integrin defense
Source: Front Microbiol. 2026 Jan 9;16:1699748. doi: 10.3389/fmicb.2025.1699748 (PMC12827662; doi:10.3389/fmicb.2025.1699748)
Supplement: Supplementary file 1 [file Table_1.docx]

**Table S1. shRNA Sequences.**

| **ShRNAs (mouse)** | **Sequence** |
| --- | --- |
| sh-ITGAM#1 | 5'- GCAGCCAGATTGGCTCTTATT -3' |
| sh-ITGAM#2 | 5'- CGCCTCAATTATACACTGGTT -3' |
| sh-ITGAM#3 | 5'- GCCTTGTGTCATGGCTTCAAT -3' |
| sh-ITGB2#1 | 5'- CCTACTATAAACTCTCCTCTA -3' |
| sh-ITGB2#2 | 5'- CGGAAGGAACATTTACAACAT -3' |
| sh-ITGB2#3 | 5'- CCAGGAATGCACCAAGTACAA -3' |
| sh-NC | 5'-TTCTCCGAACGTGTCACGT-3' |
