# Supplementary material for: Gut microbiota dysbiosis aggravates sepsis-induced lung injury by promoting neutrophil extracellular traps and suppressing host integrin defense
Source: Front Microbiol. 2026 Jan 9;16:1699748. doi: 10.3389/fmicb.2025.1699748 (PMC12827662; doi:10.3389/fmicb.2025.1699748)
Supplement: Supplementary file 2 [file Table_2.docx]

**Table S2. RT-qPCR Primer Sequences.**

| **Gene** | **Primer Sequence** |
| --- | --- |
| ITGAM (mouse) | F: 5'- ATGGACGCTGATGGCAATACC -3' |
|  | R: 5'- TCCCCATTCACGTCTCCCA -3' |
| ITGB2 (mouse) | F: 5'- TGCCGCATTCAATGTGACTTT -3' |
|  | R: 5'- CTTCTTGACGTTGTTGAGGTCAT -3' |
| GAPDH (mouse) | F: 5'- AGGTCGGTGTGAACGGATTTG -3' |
|  | R: 5'- TGTAGACCATGTAGTTGAGGTCA -3' |

Note: F: Forward, R: Reverse.
