# Supplementary material for: Gut microbiota dysbiosis aggravates sepsis-induced lung injury by promoting neutrophil extracellular traps and suppressing host integrin defense
Source: Front Microbiol. 2026 Jan 9;16:1699748. doi: 10.3389/fmicb.2025.1699748 (PMC12827662; doi:10.3389/fmicb.2025.1699748)
Supplement: Supplementary file 3 [file Table_3.docx]

**Table S3. Details of the First Antibody Product.**

| **Name** | **Cat.** | **DiIution ratio** | **Manufacturer** | **Country** | **MW (kDa)** |
| --- | --- | --- | --- | --- | --- |
| ITGAM | ab128797 | 1:1000 | Abcam | UK | 127 |
| ITGB2 | SAB5701152 | 1:500-1:2000 | Sigma-Aldrich | Germany | 100 |
| β-catenin | 71-2700 | 1:125-1:250 | Invitrogen | USA | 92 |
| ZO-1 | 61-7300 | 1:250-1:25000 | Invitrogen | USA | 225 |
| VE-cadherin | 36-1900 | 1:250 | Invitrogen | USA | 125 |
| GAPDH | ab9485 | 1: 2500 | Abcam | UK | 37 |
