# Supplementary material for: Gut microbiota dysbiosis aggravates sepsis-induced lung injury by promoting neutrophil extracellular traps and suppressing host integrin defense
Source: Front Microbiol. 2026 Jan 9;16:1699748. doi: 10.3389/fmicb.2025.1699748 (PMC12827662; doi:10.3389/fmicb.2025.1699748)
Supplement: Supplementary file 4 [file Table_4.docx]

**Table S4. Survival rate of each group of mice**

| **Group** | **Survival rate** |
| --- | --- |
| Control | 100% |
| Model | 100% |
| FMT | 100% |
| FMT+saline | 100% |
| FMT+DNase | 100% |
| FMT+sh-NC | 100% |
| FMT+sh-ITGAM | 100% |
| FMT+sh-ITGB2 | 100% |
